# Socioeconomic inequality of unintended pregnancy in the Iranian population: a decomposition approach

**DOI:** 10.1186/s12889-018-5515-5

**Published:** 2018-05-09

**Authors:** Reza Omani-Samani, Mostafa Amini Rarani, Mahdi Sepidarkish, Esmaeil Khedmati Morasae, Saman Maroufizadeh, Amir Almasi-Hashiani

**Affiliations:** 1 Department of Epidemiology and Reproductive Health, Reproductive Epidemiology Research Center, Royan Institute for Reproductive Biomedicine, ACECR, P.O. Box: 16635148, Tehran, Iran; 2 Social Determinants of Health Research Center, Isfahan University of Medical Sciences, Isfahan, Iran; 3 Department of Health Services Research, National Institute for Health Research Collaboration for Leadership in Applied Health Research and Care North West Coast (NIHR CLAHRC NWC), Institute of Psychology, Health, and Society, University of Liverpool, Liverpool, UK

**Keywords:** Unintended pregnancy, Socioeconomic inequality, Concentration index, Decomposition, Iran

## Abstract

**Background:**

There are several studies regarding the predictors or risk factors of unintended pregnancy, but only a small number of studies have been carried out concerning the socio-economic factors influencing the unintended pregnancy rate. This study aimed to determine the socioeconomic inequality of unintended pregnancy in Tehran, Iran, as a developing country.

**Methods:**

In this hospital based cross-sectional study, 5152 deliveries from 103 hospitals in Tehran (the capital of Iran) were included in the analysis in July 2015. Socioeconomic status (SES) was measured through an asset-based method and principal component analysis was carried out to calculate the household SES. The concentration index and curve was used to measure SES inequality in unintended pregnancy, and then decomposed into its determinants. The data was analyzed by statistical Stata software.

**Results:**

The Wagstaff normalized concentration index of unintended pregnancy (− 0.108 (95% Confidence Interval (CI) = − 0.119 ~ − 0.054)) endorses that unintended pregnancy is more concentrated among poorer mothers. The results showed that SES accounted for 27% of unintended pregnancy inequality, followed by the mother’s nationality (19%), father’s age (16%), mother’s age (10%), father’s education level (7%) and Body Mass Index (BMI) groups (5%).

**Conclusion:**

Unintended pregnancy is unequally distributed among Iranian women and is more concentrated among poor women. Economic status had the most positive contribution, explaining 27% of inequality in unintended pregnancy.

## Background

Unintended pregnancy is a major public health concern, being defined as a mistimed, unplanned or unwanted pregnancy at the time of conception [1]. Roughly, 213 million pregnancies took place globally in 2012, with 85% of all pregnancies occurring in developing or less developed countries, 56% of which occurred in Asia [2]. A proportion of all pregnancies are unintended or unplanned. In 2012, the worldwide rate of unintended pregnancy was 53 cases per 1000 women aged 15 to 44 years old. Bases on the data reported in 2012, 40% of pregnancies were unintended, a rate which has not significant decreased in recent decades (in 1995 and 2008 it was 43 and 42%, respectively) [2, 3]. In the United State, it was reported that 49% of pregnancies in 2006 and 48% in 2001 were unintended [4]. In another study conducted in United States, it was reported that 45% of pregnancies in 2011 and 51% in 2008 were unintended [5].

Based on a meta-analysis study in Iran, the unwanted pregnancy rate is 30.6% [6]. Even with the government’s efforts regarding family planning, nearly 55.4% of women at reproductive age use contraceptive methods [7].

A large number of studies have been conducted to detect the unintended pregnancy rate, its predictors and outcomes. For example, several studies have suggested that unintended pregnancies have some adverse health, socioeconomic and psychological outcomes for both women and their children [8–12]; however, only a small number of studies have been carried out concerning socio-economic factors influencing the unintended pregnancy rate.

The social determinants of health have an important impact on unintended pregnancy. In some studies [13–15], it has been shown that lower educational attainment is strongly associated with unplanned pregnancy. Controversially, Hamdela et al. [16] attested that there is no association between maternal education and unintended pregnancy. On the other hand, previous studies have suggested that unemployment has a significant association with unintended pregnancy [17, 18]. Goossens et al. [14] concluded that less-planned pregnancies were associated with lower socio-economic status. Briefly, there is some evidence that unintended pregnancy is associated with SES.

To the best of our knowledge, there has been no study quantitatively examining the impact of socio-economic inequality on unintended pregnancy. In the current study, we want to clarify the socioeconomic inequality in unintended pregnancy in Tehran, the capital of Iran, by means of a concentration index (CI) decomposition approach. This method helps to identify the major predictors of socioeconomic inequalities in unintended pregnancy in societies, which will be important for health policy makers. Therefore, this study aimed to determine the socioeconomic inequality in unintended pregnancy in Tehran, Iran, as a developing country.

## Methods

### Design, setting, and patients

In this hospital based cross-sectional study, 5152 mothers in labor who were referred to the Obstetrics and Gynecology Wards of 103 hospitals (affiliated with Tehran, Beheshti, and Iran’s Universities of Medical Sciences) in Tehran province (Capital of Iran) were included in the analysis.

We included all women in the defined period (between 6th to 21st July, 2015) in this study, regardless of the type of delivery (natural or cesarean section), the pregnancy outcome (live birth, stillbirth, or spontaneous abortion), type of hospitals (private or governmental), gravidity (nulliparous or multiparous), and singleton or twin. All women who gave informed consent were included in the study. The required data was gathered from medical centers with obstetrics and gynecology wards. The sampling process was performed for two weeks and the data was collected by 103 trained midwives or nurses. More detail about methodology was reported elsewhere [19–24].

### Variable definition

Unintended pregnancy was selected as a dichotomous outcome variable, i.e. whether each of the interviewed mothers had experienced an unplanned pregnancy or not. However, and most importantly, the socioeconomic status (SES) of participants was measured using an asset based approach. In fact, there are three approaches to measure socioeconomic status: income, expenditure, and assets. Income estimation is the direct method for measurement of socioeconomic status, but as researchers usually have no access to the people’s true income, especially in developing countries, the income method is not normally suggested. The expenditure method is also not suggested as a favored method, as modern human life entails a plethora of expenditure items, ranging from health, food, housing, education, to recreational expenditures. To put it better, this enaction of this method consumes a vast amount of both time and resources. However, economists have suggested that the possession of household assets can be used as a proxy for income and expenditure as a socioeconomic measure. In the present study, to assess the SES via the asset-based method, the pregnant women were asked about whether they possessed certain assets, including a laptop, freezer, dish washing machine, vacuum cleaner, handicraft carpet, private cars, three-dimensional TV, side by side refrigerator, smart phone, a microwave, the number of rooms in their house, and the area of their residence per meter. Principal Component Analysis (PCA) was used to determine a household’s economic status, based on their possession of the above-mentioned assets [25].

Among the determinant variables, BMI values were categorized into different BMI groups, as previously described [26]: underweight (BMI < =19.9), normal (BMI = 20–24.9) overweight (BMI = 25–29.9) obese (BMI = 30–39.9) and morbidly obese (BMI > =40).

### Statistical analysis

Inequality in unintended pregnancy was explored through the use of a Concentration Index (CI) [27]. As a CI is a decomposable index, it is widely when measuring inequality in health [28]. The CI was calculated as twice the covariance of a health variable and the fractional rank of a socioeconomic variable divided by the mean of a health-related variable, as follows:

1 $$ \mathrm{C}=\frac{2}{\mathrm{n}\upmu}{\sum}_{\mathrm{i}=1}^{\mathrm{n}}{\mathrm{y}}_{\mathrm{i}}{\mathrm{R}}_{\mathrm{i}}-1 $$

Where y_i_ denotes the health variable (i.e. unintended pregnancy) of i th individual, μ indicates its mean and R_i_ denotes the fractional rank of i th individual in terms of the index of their socio-economic status. CI values can vary between −1 and + 1; the negative and positive values indicated that inequality is disproportionally concentrated in either the poor or the rich (pro-rich and pro poor inequality), respectively, as well the value being zero in the case of no inequality [27]. Since, in this study, unintended pregnancy is a dichotomous variable, a normalization of the CI was needed to calculate inequality properly [29]. Wagstaff has suggested that the index could be normalized as follow [30]:

2 $$ {\mathrm{C}}_{\mathrm{normalized}}=\frac{\mathrm{c}}{1-\upmu}\kern5.25em $$

Therefore, in this study we used Wagstaff’s normalized concentration index (WCI) to measure and decompose unintended pregnancy inequality.

### Decomposition approach

To reveal the contribution of each explanatory variable to the measured health inequality (i.e. unintended pregnancy inequality), a CI decomposition approach was used. According to Wagstaff, van Doorslaer and Watanabe [31], we applied a linear regression model linking unintended pregnancy (y) to a set of k determinants (x_k_):

3 $$ {\mathrm{y}}_{\mathrm{i}}=\upalpha +\sum \limits_{\mathrm{k}}{\upbeta}_{\mathrm{k}}{\mathrm{x}}_{\mathrm{k}\mathrm{i}}+{\upvarepsilon}_{\mathrm{i}}\kern3.5em $$

Where${\mathrm{x}}_{{\mathrm{k}}_{\mathrm{i}}}$ is a set of k determinant variables for the i th individual, β_k_ signifies the coefficient, and ε_i_ is an error term. Given the association of y_i_ and ${\mathrm{x}}_{{\mathrm{k}}_{\mathrm{i}}}$ in eq. (3), CI for (y) can be represented as:

4 $$ \mathrm{C}=\sum \limits_{\mathrm{k}}\left(\frac{\upbeta_{\mathrm{k}}{\overline{\mathrm{x}}}_{\mathrm{k}}}{\upmu}\right){\mathrm{C}}_{\mathrm{k}}+\frac{\mathrm{G}{\mathrm{C}}_{\upvarepsilon}}{\upmu}={\mathrm{C}}_{\widehat{\mathrm{y}}}+\frac{\mathrm{G}{\mathrm{C}}_{\upvarepsilon}}{\upmu}\kern3.75em $$

Where is μ the mean of y, ${\overline{\mathrm{x}}}_{\mathrm{k}}$ is the mean of x_k_, C_k_ is the normalized concentration index for x_k_ (defined exactly like the CI for unintended pregnancy), $\frac{\upbeta_{\mathrm{k}}{\overline{\mathrm{x}}}_{\mathrm{k}}}{\upmu}$ is the elasticity of unintended pregnancy with explanatory variables, and GC_ε_ is the generalized CI for ε_i_ (residual component). More details pertaining to the CI decomposition method have been provided elsewhere [27, 31]. The first step in conducting the decomposition analysis is running an appropriate regression model for calculating the coefficients (β_k_) of the explanatory variables. Taking account of the dichotomous nature of unintended pregnancy in this study, and following Yiengprugsawan et al., a Generalized Linear Model (GLM) (with a binomial family and identity link) [32] was used for decomposing unintended pregnancy inequality. The pros of GLM, when compared to other regression models, is that it leads to valid coefficient estimates that do not differ by choice of reference group ( [33]). Data analysis was carried out using Stata statistical software (Stata version 13.0; Stata Corp LP, College Station, TX Stata).

## Results

We analyzed 5152 deliveries in July 2015. Of these 20% were unintended pregnancy (*n* = 1021). 40.02 and 35.50% of mothers and fathers had a diploma, respectively. Based on the BMI categories, most of the mothers (43.25%) had a normal weight (BMIs were in the range of 20–24.9). Most of the mothers and fathers were aged 20–39 and 30–39, respectively. Fortunately, most of the mothers didn’t have a history of abortion (80%) or miscarriage (99%). The results show that 42.08% of the studied mothers had been pregnant at least four times (Table 1).

**Table 1:** Summary statistics about unintended pregnancy and its determinants in Iran, (2015)

Variable			
		n	**%**
Unintended pregnancy	Yes	1021	19.82
	No	4131	80.18
Scio-economic status	Poorest	1063	20.63
	Poorer	1048	20.34
	Middle	1006	19.53
	Richer	1018	19.76
	Richest	1017	19.74
Mother’s education level	Illiterate	204	3.95
	under Diploma	1219	23.67
	Diploma	2062	40.02
	Academic	1667	32.36
Father’s education level	Illiterate	164	3.18
	under Diploma	1534	29.77
	Diploma	1828	35.50
	Academic	1626	31.55
BMI category	Underweight	550	10.68
	Normal	2228	43.25
	Overweight	1623	31.50
	Obese	535	10.38
	Morbidly obese	216	4.19
Mother’s age	<=19	180	3.49
	20–29	2414	46.86
	30–39	2375	46.10
	> = 40	183	3.55
Father’s age	<=19	4	0.08
	20–29	1237	24.01
	30–39	3089	59.96
	> = 40	822	15.95
Mother’s nationality	Iranian	4788	92.93
	Non- Iranian	364	7.07
History of abortion	Have	1033	20.05
	Not have	4119	79.95
History of miscarriage	Have	39	0.75
	Not have	5113	99.25
Number of pregnancy	0	1520	29.50
	1–3	1464	28.42
	> = 4	2168	42.08

**Fig. 1:**
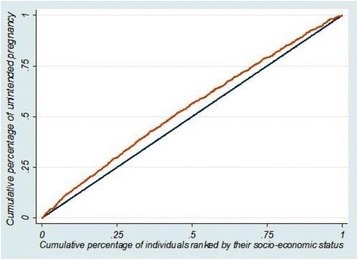
Concentration curve of unintended pregnancy in Iran (2015)

Figure 1 illustrates the concentration curve of unintended pregnancy. As Fig. 1 depicts, the concentration curve of unintended pregnancy lay above the line of equality. This means that unintended pregnancy was more concentrated among the poorer participants. To be exact, this reveals that there is an unequal disfavoring of the poor in unintended pregnancy in Iran. Besides, the WCI value of unintended pregnancy (− 0.108 (95% CI = − 0.119 ~ − 0.054)) endorses that unintended pregnancy is more concentrated among poorer mothers (Table 2).

**Table 2:** Wagstaff normalized concentration index (WCI) (95% confidence interval, standard error and *P*-value) for unintended pregnancy in Iran

Unintended pregnancy	WCI value	Std. Err.	95% Conf. interval for CI	*P*-value
	−0.108	0.020	− 0.119 ~ − 0.054	< 0.001

The decomposition of unintended pregnancy is shown in Table 3. The WCIs of the determinant variables show that a number of features were concentrated among study participants with a lower socioeconomic status, such as the mother and father having a low educational level, being underweight, overweight or obese, aged < 29 years, and having experienced a larger number of pregnancies and deliveries. As the table shows, SES (about 27%) accounted for most of the unintended pregnancy inequality. If the value of the contribution of variable X were x and positive (negative), then, if the variable were to be equally distributed across the different socioeconomic groups, the unintended pregnancy inequality would decrease (increase) by x%. So, if socioeconomic resources were equally distributed across different SES groups, then inequality in unintended pregnancy in Iran would decrease by up to 27%. Mother nationality (19%), father’s age (16%), mother’s age (10%), Father’s education level (7%) and BMI groups (5%) followed in terms of the importance of their contribution to unintended pregnancy inequality in Iran. Table 3 also displays the residual components. The overall WCI of unintended pregnancy was − 0.108. The observed component of the overall WCI was − 0.085. This component shows that the determinants included in the current model were able to explain 79% of the measured inequality in unintended pregnancy in Iran. The rest of the inequality was due to residual (20.93%) components.

**Table 3:** Decompositions of concentration index for unintended pregnancy in Iran

	coefficient	Elasticity	normalized C_k_	Absolute contribution	Percent Contribution
SES					
The poorest	0.018	−0.019	−1.000	− 0.019	17.59
Poorer	0.017	0.016	−0.481	−0.008	7.34
Middle	−0.029	−0.029	0.002	0.000	0.05
Richer	−0.004	−0.004	0.500	−0.002	1.87
The richest*	–	–	–	–	–
Sum					26.86
Mother’s education level					
Illiterate	0.001	0.000	−0.797	0.000	0.15
Under diploma	−0.001	−0.001	− 0.518	0.001	− 0.57
Diploma	0.008	0.016	−0.054	−0.001	0.81
Academic *	–	–	–	–	–
Sum					0.39
Father’s education level					
Illiterate	0.006	0.001	−0.739	−0.001	0.64
Under diploma	0.009	0.014	−0.551	−0.007	6.89
Diploma	0.011	0.020	0.017	0.000	−0.31
Academic*	–	–	–	–	–
Sum					7.22
BMI category					
Underweight	−0.062	−0.031	− 0.143	0.004	−4.06
Normal	−0.045	−0.099	0.115	−0.011	10.50
Overweight	−0.092	−0.150	− 0.005	0.001	− 0.69
Obese	−0.049	− 0.025	−0.020	0.001	−0.47
Morbidly obese*	–	–	–	–	–
Sum					5.28
Mother’s age					
<=19	0.017	0.003	−0.350	−0.001	0.97
20–29	−0.051	−0.120	− 0.153	0.018	−17.04
30–39	−0.061	− 0.142	0.201	− 0.029	26.43
> = 40*	–	–	–	–	–
Sum					10.37
Father’s age					
<=19	0.04	0.000	−0.607	0.000	0.11
20–29	−0.035	−0.042	− 0.224	0.010	−8.80
30–39	−0.074	− 0.223	0.121	− 0.027	25.04
> = 40*	–	–	–	–	–
Sum					16.35
Mother’s nationality					
Iranian	−0.016	−0.075	0.276	−0.021	19.18
Others*	–	–	–	–	–
History of abortion					
Yes	0.016	0.016	0.035	0.001	−0.52
No*	–	–	–	–	–
History of miscarriage					
Yes	−0.056	−0.002	0.177	0.000	0.32
No*		–	–	–	–
Number of pregnancy					
0	0.042	0.062	0.120	0.007	−6.93
1–3	0.011	0.016	−0.038	−0.001	0.56
> = 4*	–	–	–	–	–
Sum					−6.37
Total observed				−0.085	79.07
Residual				−0.023	20.93
Total				−0.108	100

*denotes reference group

## Discussion

The results of present study show that the concentration curve of unintended pregnancy lies above the line of equality in the concentration curve. This means that unintended pregnancy is more concentrated among the poor and there is an unequal disfavoring of the poor in unintended pregnancy in Iran. Then, in order to disclose the contribution of each independent variable to the measured unintended pregnancy inequality, a concentration index decomposition approach was used. The results showed that SES accounted for 27% of unintended pregnancy inequality, followed by mother’s nationality (19%), father’s age (16%), mother’s age (10%), father’s education level (7%) and BMI groups (5%). If the value of the contribution of variable X were x and positive (negative), then unintended pregnancy inequality would decrease (increase) by x% if the variable were to be equally distributed across different socioeconomic groups. For as much as SES accounted for 27% of the unintended pregnancy inequality, if socioeconomic resources were equally distributed across the different SES groups, inequality in unintended pregnancy could decrease by up to 27% in Iran. Also, the results showed that the determinants included in the current model were able to explain 79% of the measured inequality in unintended pregnancy in Iran. However, in this study, 21% of unintended pregnancy inequality could not be explained by the systematic variation in the determinant variables across socioeconomic groups.

Unintended pregnancy is one of the most worrying public health issues and an extremely important worldwide reproductive health concern, leading to a considerable socioeconomic burden on both persons and wider communities [34]. There are several studies regarding the predictors or risk factors of unintended pregnancy [15, 35–38] as well as studies that explore the relationship between socioeconomic inequality and unintended pregnancy [39, 40], but such studies used traditional modes of analysis, such as logistic regression and odds ratio, whereas the concentration index and concentration curve methodology has been recommended specifically for the measurement of economic inequality in health outcomes [27, 30–33, 41]. Also, in previous studies of a similar nature [39], only educational level was used as an indicator of socioeconomic position, whereas the measure of household wealth can be assessed through multiple channels, such as “income level”, “consumption or expenditure data” and “asset-based data”. In this study we used the asset-based method as it is more appropriate for the study of developing countries. The asset-based technique is a fast and uncomplicated approach for collecting economic status data. This is because the collection of data requires little time [42, 43] and also because the asset based method is a more stable measure of economic status than consumption expenditure when considering factors such as economic shock or change [43].

No previous study has addressed the question of unintended pregnancy inequality through an approach comparable to the present study. Based on our results, we can state that unintended pregnancy was more concentrated among the poor and that SES accounted for 27% of unintended pregnancy inequality. This result has been confirmed by other published studies. Font-Ribera et al. [39] have shown that there is socioeconomic inequality in unintended pregnancy in Barcelona, Spain, and that unintended pregnancy is more concentrated among women in low socioeconomic positions. As previously stated, educational level was used as an indicator of socioeconomic position in Font-Ribera et al’s study, in which they demonstrated that women with an elementary education had higher odds of experiencing unintended pregnancies (7.22 times) than those with an university education.

The results of Lawrence Ikamari et al’s [44] study indicate that education level is not related with the incidence of unintended pregnancy. However, after adjusting their confounder variables (education, wealth index, employment status, ethnicity, household size and residence), household wealth index was significantly associated with unintended pregnancy, with women from medium (odds ratio = 0.66) and rich households (odds ratio = 0.51) being less likely to experience an unintended pregnancy than from those poor households. Employed women were also less likely to experience unintended pregnancy than unemployed women. In our study, the mother’s education exhibited no meaningful contribution to unintended pregnancy inequality. Finer and Zolna’s study [4] revealed that women with a low education level had the maximum unintended pregnancy rate, and that, in the case of economic status, the unintended pregnancy rate among deprived women was more than five times the rate for women in the highest income level. Therefore, our finding is consistent with other studies that report the association between economic status and a higher risk of unintended pregnancy [4, 39, 44].

### Strengths and limitations of this study

In this study we measured economic status using an asset-based method, which is quick, simple and precise compared to other methods. Instead of using traditional analysis, for the first time, we used concentration index and concentration curve analysis to measure economic inequality and then decomposed the measured inequality. This is a cross sectional study with a self-administered questionnaire, therefore a causal interpretation of result should regarded with caution. Also, due to the cross sectional design, some biases may have been induced.

## Conclusion

In conclusion, unintended pregnancy is unequally distributed among the Iranian women and is more concentrated among poor women. Economic status has the most positive contribution, explaining 27% of inequality in unintended pregnancy.

## Abbreviations

BMI: Body mass index
CI: Concentration Index
CI: Confidence interval
GLM: Generalized Linear Model
PCA: Principal Component Analysis
SES: Socioeconomic status
WCI: Wagstaff normalized concentration index
